# A rapid and label-free platform for virus capture and identification from clinical samples

**DOI:** 10.1073/pnas.1910113117

**Published:** 2019-12-27

**Authors:** Yin-Ting Yeh, Kristen Gulino, YuHe Zhang, Aswathy Sabestien, Tsui-Wen Chou, Bin Zhou, Zhong Lin, Istvan Albert, Huaguang Lu, Venkataraman Swaminathan, Elodie Ghedin, Mauricio Terrones

**Affiliations:** ^a^Department of Physics, The Pennsylvania State University, University Park, PA 16802;; ^b^Department of Biology, New York University, New York, NY 10003;; ^c^Department of Biochemistry and Molecular Biology, The Pennsylvania State University, University Park, PA 16802;; ^d^Department of Veterinary and Biomedical Sciences, The Pennsylvania State University, University Park, PA 16802

**Keywords:** carbon nanotube, microfabrication, infectious disease, sequencing, virus isolation

## Abstract

Emerging and reemerging viruses are responsible for a number of recent epidemic outbreaks. A crucial step in predicting and controlling outbreaks is the timely and accurate characterization of emerging virus strains. We present a portable microfluidic platform containing carbon nanotube arrays with differential filtration porosity for the rapid enrichment and optical identification of viruses. Different emerging strains (or unknown viruses) can be enriched and identified in real time through a multivirus capture component in conjunction with surface-enhanced Raman spectroscopy. More importantly, after viral capture and detection on a chip, viruses remain viable and get purified in a microdevice that permits subsequent in-depth characterizations by various conventional methods. We validated this platform using different subtypes of avian influenza A viruses and human samples with respiratory infections. This technology successfully enriched rhinovirus, influenza virus, and parainfluenza viruses, and maintained the stoichiometric viral proportions when the samples contained more than one type of virus, thus emulating coinfection. Viral capture and detection took only a few minutes with a 70-fold enrichment enhancement; detection could be achieved with as little as 10^2^ EID_50_/mL (50% egg infective dose per microliter), with a virus specificity of 90%. After enrichment using the device, we demonstrated by sequencing that the abundance of viral-specific reads significantly increased from 4.1 to 31.8% for parainfluenza and from 0.08 to 0.44% for influenza virus. This enrichment method coupled to Raman virus identification constitutes an innovative system that could be used to quickly track and monitor viral outbreaks in real time.

## Significance

Viruses evolve rapidly and unpredictably, challenging the effectiveness of disease diagnostics. To help control outbreaks and understand their origins, the first step is often isolating viruses from infected samples for characterization. We demonstrate that multiple emerging virus strains can be simultaneously enriched and optically detected in only a few minutes without using any labels. A portable platform that captures viruses by their size, coupled to Raman spectroscopy, resulted in successful virus identification with 90% accuracy in real time directly from clinical samples. Furthermore, this viable enrichment process enables further culturing and characterization by electron microscopy and deep sequencing. This microplatform is an effective disease-monitoring system and broadens virus surveillance by enabling real-time virus identification.

Viruses are the most abundant biological entities on earth (1), more than archaea and bacteria combined. They also represent the largest and most genetically diverse reservoir of nucleic acid that can infect all forms of life (2–4). Viruses can evolve rapidly and cause new epidemics, such as the zoonotic H5N1 pandemic (5, 6), Zika (7), and the recent Ebola outbreaks (8, 9). Studies covering the period of the last century have shown that globalization and industrialization played a vital role in the emergence and dissemination of viral diseases (9–11). Currently, 1.67 million unknown viruses are estimated to be circulating in animal reservoirs, a number of which with the potential for zoonotic transmission to humans (12). Therefore, a better understanding of circulating viruses and their epidemiology is critical for better preparedness (3, 12–16). Closely monitoring zoonotic virus strains, as well as rapidly identifying unknown viruses, would aid in preparation for future outbreaks. According to the World Health Organization, early detection can halt virus spread by enabling the rapid deployment of proper countermeasures (17, 18). Methods for outbreak detection have benefitted from new genomic tools. However, effective virus surveillance and discovery remain challenging (3, 9, 12, 15, 19, 20).

In virus surveillance, collected samples are subjected to a series of time-consuming steps, such as ultracentrifugation and cell culture, to enrich virus particles or amplify virus titers (3, 14, 21–23). In addition, many viruses are not easily culturable, and bias is often introduced during amplification, leading to artifacts in the sequence data (14, 24). Even standard methods require a laboratory with proper infrastructure and are time intensive, ranging from several hours to days. Unfortunately, such methodologies for viral enrichment are impractical when samples are time-sensitive or have variable viral titers.

Existing technologies, such as immune-based (25) and molecular assays (13) [e.g., enzyme-linked immunosorbent assay (ELISA) (26) and PCR (27)], provide relatively sensitive detection for the identification of viruses but require prior knowledge of the strains. Deep sequencing techniques, such as next-generation sequencing (NGS) (14, 23), are powerful tools in virus surveillance. NGS can detect mutations in virus genomes and capture information on genetic diversity within the virus population if sequence coverage is sufficient. One current challenge is the low virus titer in most samples, leading to sequence reads that are dominated by host genetic material rather than by viral pathogens. Extant enrichment methods, including virus culture and genome amplification, often introduce artificial variants or bias in the sequence reads. In addition, these processing steps also involve incorporating different benchtop equipment, reagents, and technical expertise during operation. Thus, rapid enrichment and fast virus detection in remote field conditions remain a critical technical hurdle for outbreak preparedness (3, 9, 12, 15, 19, 20).

We have previously shown that viruses can be captured between aligned carbon nanotubes, without the use of specific labels, when their sizes match the intertubular distance (ITD) (28). In our previous iteration, we assembled a simple filtration device to perform size-based capture of individual types of viruses. In addition, the processing speed of the virus samples was low at approximately 1 mL per h, and not suitable for clinical samples due to clogging from tissue or cell debris. In this report, we present a complete and high-throughput sample preparation platform, named VIRRION (virus capture with rapid Raman spectroscopy detection and identification), for rapid multivirus enrichment and label-free detection directly from clinical samples. VIRRION consists of a handheld microdevice, designed to simultaneously capture different viruses by size while preserving their structural integrity and viability, and to perform real-time nondestructive identification using surface-enhanced Raman spectroscopy (SERS) coupled to a machine learning algorithm and database. We demonstrate that VIRRION can be used to successfully capture, enrich, and optically identify unknown and highly mutated strains from clinical samples without minimal sample manipulation. More importantly, this nondestructive approach allows follow-up analyses using more conventional methods for virus characterization, including virus isolation, immunostaining, and sequencing.

## Results

### Design and Assembly of the VIRRION Platform.

**Fig. 1.**
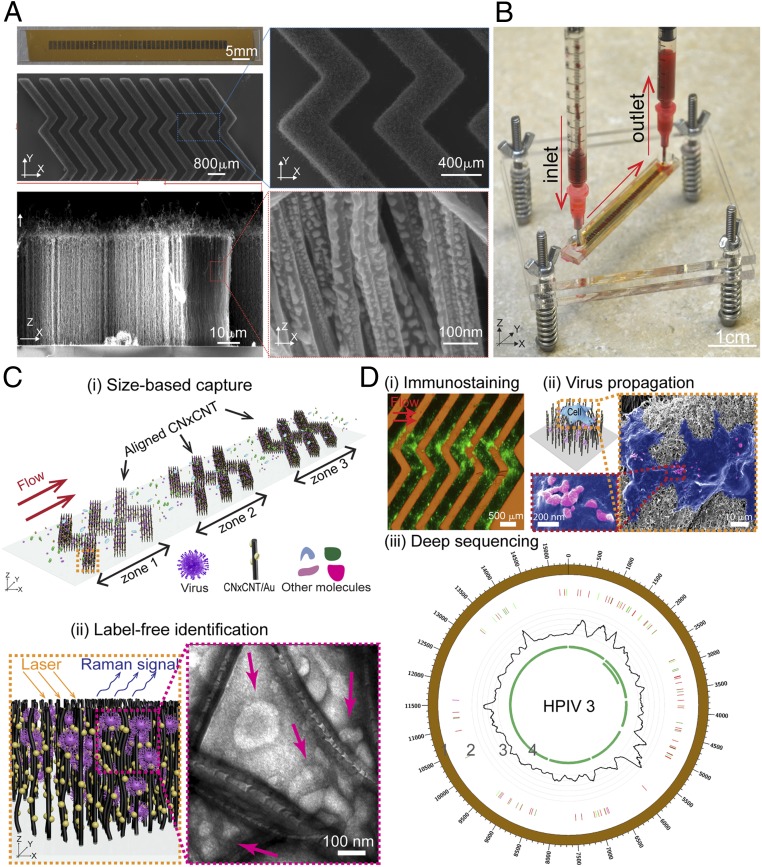
Design and working principle of VIRRION for effective virus capture and identification. (*A*) Photograph and SEM images of aligned CNTs exhibiting herringbone patterns decorated with gold nanoparticles. (*B*) Picture showing assembled VIRRION device, processing a blood sample. (*C*) Illustration of (*i*) size-based capture and (*ii*) in situ Raman spectroscopy for label-free optical virus identification. Images of electron microscopy showing captured avian influenza virus H5N2 by CNxCNT arrays. (*D*) On-chip virus analysis and enrichment for NGS, (*i*) on-chip immunostaining for captured H5N2, (*ii*) on-chip viral propagation through cell culture, and (*iii*) genomic sequencing and analysis of human parainfluenza virus type 3 (HPIV 3). Track 1: scale of the base pair position; track 2: variant analysis by mapping to strain #MF973163; color code: deletion (black), transition (A–G, fluorescent green; G–A, dark green; C–T, dark red; T–C, light red), transversion (A–C, brown; C–A, purple; A–T, dark blue; T–A, fluorescent blue; G–T, dark orange; T–G, violet; C–G, yellow; G–C, light violet); track 3: coverage; track 4: regions of open reading frames (ORFs).

The VIRRION platform is constructed with aligned nitrogen-doped carbon nanotube (CNxCNT) arrays, decorated with gold (Au) nanoparticles to separate, enrich, and detect fluidic human samples by direct Raman-based spectroscopic identification (Fig. 1*A* and *SI Appendix*, Fig. S1). In our previous study, we demonstrated that the CNxCNTs had better biocompatibility than the pristine CNTs (28). For VIRRION, CNxCNTs were used as the building blocks, but we added a stamping technique to pattern Fe catalytic particles. After chemical vapor deposition (CVD), CNxCNTs grew on Fe particles and formed aligned nanotube arrays. They were then coated with Au nanoparticles (Au/CNxCNTs) to enhance the signal-to-noise ratio in Raman spectroscopy. This stamping method allowed us to pattern Fe catalytic particles with a concentration gradient using a simple and low-cost process, compared to a conventional lithography-based fabrication technique that requires a range of equipment to obtain the same pattern involving a variety of chemicals and photoresists, as well as several steps of patterning (including lithography and deposition) that have to be repeated sequentially. Using this process, multiple zones of the herringbone arrays with different ITDs, from 22 ± 5 to 720 ± 64 nm, were patterned to match viruses of different sizes. Previous studies have shown that herringbone patterns, made of PDMS (polydimethylsiloxane), enhance mixing of the samples inside a microfluidic channel by inducing a chaotic flow (29, 30). We incorporated the herringbone patterns to leverage enhanced mixing with the aligned Au/CNxCNTs. The 3-dimensional (3D) and porous herringbone pattern enhanced the interactions between the viruses and the Au/CNxCNT arrays, and the presence of a gradient of ITDs selectively captured different particles based on size. While our previous report demonstrated the feasibility of a size-based virus capture approach (28), the study presented here is on the design and fabrication of a microplatform for the rapid capture of viruses, and the validation with conventional methods, such as electron microscopy, immunostaining, virus isolation through cell culture, and NGS (Fig. 1 *C* and *D*).

### Stamping Technique for Patterning CNxCNT Arrays and In Situ Raman Spectroscopy.

**Fig. 2.**
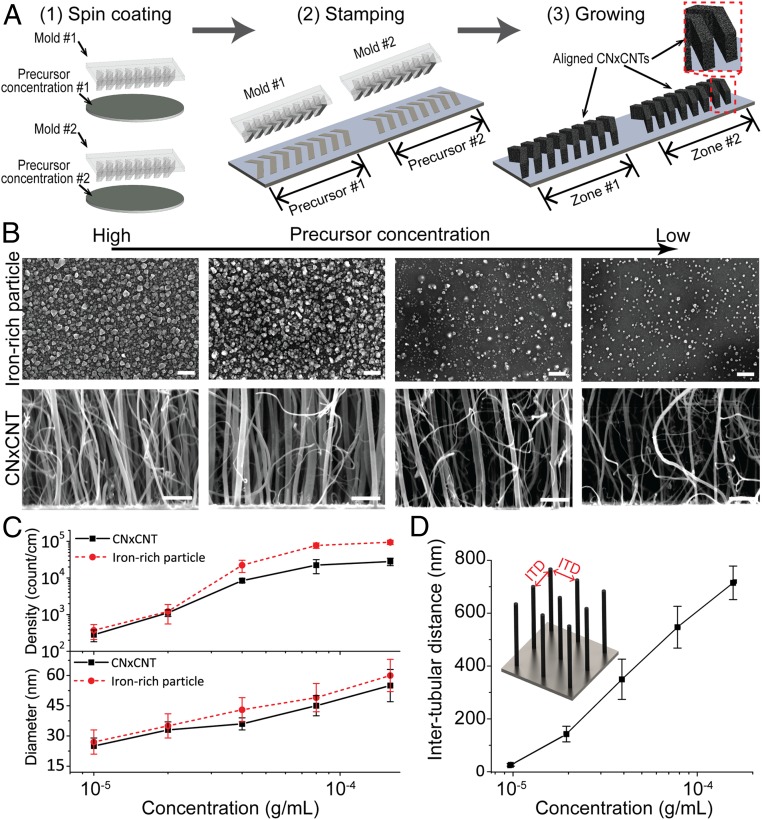
Stamping technique for aligned CNxCNT growth with tunable dimensions. (*A*) Process flow of the stamping technique for patterning CNxCNT arrays. (*B*) SEM of iron-rich particles (top row) and CNxCNT (bottom row) before and after CVD synthesis. [Scale bar: 100 nm (top row); 100 nm (bottom row).] (*C*) Density and diameter of iron-rich particles and CNxCNTs under different precursor concentrations. (*D*) Tunable ITD of aligned CNxCNT array growing under different precursor concentrations.

In our previous study, we applied an expensive and time-consuming microlithography-based technique to selectively grow aligned CNxCNT arrays on silicon substrates (28). In this report, we expanded on this method by developing a stamping technique to easily pattern catalytic Fe particles, which are responsible for growing aligned CNxCNTs with herringbone configurations exhibiting a gradient of ITDs that match the sizes of different viruses (Fig. 2). Without using any lithography and metal deposition, we utilize a 3D printing technique to fabricate a reusable mold that patterns liquid-based Fe precursor on a substrate in several minutes with a submillimeter resolution. More importantly, our measurement showed that this stamping technique increases the range of the ITDs from 25 to 325 nm, to 22 to 720 nm. This expanded range covers a large diversity of virus particle sizes, thus making possible size-based and label-free capture of different viruses without the need for prior information about the virus strain. Specifically, we used a commercial 3D printer to prepare polymer micromolds with herringbone micropatterns that were used to deposit different concentration solutions of Fe precursors onto Si/SiO_2_ substrates and to grow aligned CNxCNTs with tuned ITDs (Fig. 2*A*). The technique started with spin-coating iron156(III) nitrate nonahydrate, Fe(NO_3_)_3_•9H_2_O, on micromolds with herringbone patterns. Then, Fe-based precursors were transferred on a Si/SiO_2_ substrate by stamping the side of the micromolds covered with precursors. During spin-coating and stamping, the concentration and location of the precursor were controlled and defined. As a result, herringbone-shaped precursor was applied and patterned on a substrate. The stamped precursor arrays were then used as catalytic substrates to grow arrays of aligned CNxCNTs using CVD in the presence of benzylamine (28). After CVD, aligned CNxCNTs were grown and formed 3D herringbone arrays with different ITDs in each zone. The height of the aligned CNxCNTs reached ∼70 µm after growing for 40 min. Structural characterization of the grown nanotubes revealed compartmentalized bamboo-like tubular structures containing N-dopants, as observed by high-resolution transmission electron microscopy and Raman spectroscopy (*SI Appendix*, Fig. S2 *A*–*C*) (31).

During CNxCNT CVD growth, Fe particles formed upon heating the Fe-based precursors [Fe(NO_3_)_3_•9H_2_O] (Fig. 2*B*), and the newly formed Fe particles acted as catalytic nucleation sites responsible for growing the aligned CNxCNTs (32, 33). We observed that different concentrations of Fe-based precursors resulted in Fe particles of varying density and diameter that were responsible for growing aligned CNxCNTs of different diameters and ITDs. Fig. 2*C* shows the relationship between the density and dimension of the Fe particles and CNxCNTs, and the concentration of the Fe-based precursor. By increasing the precursor concentration, the diameter and density of Fe-rich particles as well as those of CNxCNTs also increased. More importantly, we observed that the CNxCNT array has tunable ITDs that are a function of the concentration of Fe-based precursors, and the ITDs ranged from 22 ± 5 to 720 ± 64 nm (Fig. 2*D*), measured by cross-sectional scanning electron microscopy (SEM) (28). Thus, this stamping technique allows patterning a gradient of aligned CNxCNT arrays with controlled dimensions in a simple and cost-effective way. Furthermore, the tunable ITD covers the range of most virus sizes (34).

To optically detect the captured viruses using a label-free approach, we functionalized the CNxCNTs with Au nanoparticles to enable SERS (Fig. 1*A*). Raman spectroscopy is an optical spectroscopic technique that is commonly used to identify the chemical structure of substances by providing their structural/vibrational fingerprints. However, the efficiency of the Raman scattering signal is usually very weak—approximately 1 out of 10^6^ phonons is absorbed or emitted through Raman (inelastic) scattering. This weak efficiency dramatically limits the signal intensity of Raman spectra, thus constraining its application. However, studies have shown that Au nanoparticles enhance localized plasmon resonance, which can further enhance the signal of Raman scattering up to ∼10^8^. Interestingly, this Raman signal enhancement enables the detection down to a single-molecule level (approximately picomolar) (35, 36). Previous studies have demonstrated that SERS can collect fingerprints of pure viruses prepared by ultracentrifugation or by conjugating antibodies on a metal surface to isolate or capture viruses (37–39). We observed that Au nanoparticles of ∼15 nm in diameter are well distributed on CNxCNT (*SI Appendix*, Figs. S2 *C* and *D*). Results of the UV-Vis measurements indicate that the Au/CNxCNT arrays have a strong localized surface plasmon resonance (*SI Appendix*, Fig. S3*A*). We characterized the sensitivity and uniformity of the SERS detection using a reference Raman dye, Rhodamine 6G (R6G) (*SI Appendix*, Note S1 and Fig. S3*B*). The results from all of the characterizations showed that the Au/CNxCNTs arrays provide uniform, consistent, and sensitive (10^−10^ M) SERS signals across different ITDs.

### Characterization of Size-Based Capture of Virus Particles.

**Fig. 3.**
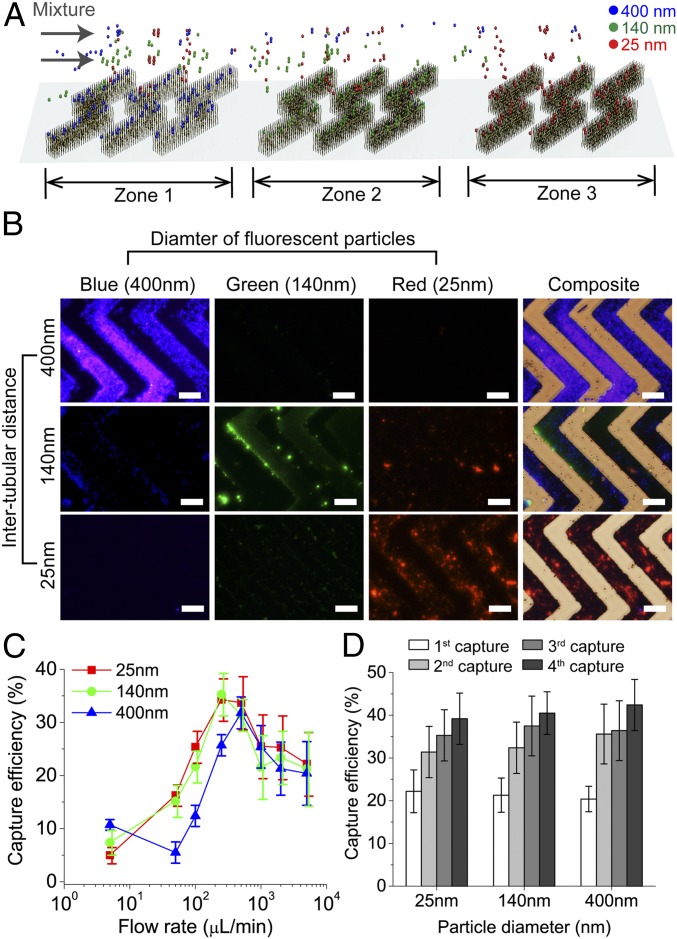
Characterization of size-based capture. (*A*) Illustration of capture and separation of 3 different sizes of fluorescently labeled silica particles by VIRRION with 3 zones of ITDs. (*B*) Fluorescent and combined bright-field images of particles captured and separated by VIRRION into individual zones. (Scale bar: 200 µm.) (*C*) Capture efficiency of different silica particles captured by VIRRIONS under different flow rates. (*D*) Capture efficiency of silica particles after multiple repeated capture by the same VIRRION.

After growing the CNxCNT arrays and fabricating microfluidic devices, we characterized the size-based capture by using different sizes of fluorescently labeled silica particles (Fig. 3*A*). We mixed together fluorescently labeled silica particles of different diameters (400, 140, and 25 nm) and of different fluorescent wavelengths (415, 508, and 612 nm, respectively; *SI Appendix*, Fig. S4 *A* and *B*). To capture and separate each subgroup of silica particles in the mixture, we assembled a microdevice with 3 zones of aligned CNxCNT arrays having 400-, 140-, and 25-nm ITDs to match their sizes. The efficiency of the size-based capture was calculated under different flow rates by measuring the fluorescent intensities of the flow through and normalizing by the initial intensities. After capture, strong fluorescent signals of individual subgroups of silica particles were detected in the CNxCNT array zones. The ITDs matched the silica bead sizes (Fig. 3*B*), illustrating clearly that VIRRION successfully captured and separated different sizes of particles from the mixture. When the flow rate ranged between 250 and 500 µL/min, the capture efficiency reached 31.8 ± 3.3%, 35.3 ± 4.7%, and 34.3 ± 4.5%, respectively, for silica particles of 400, 140, or 25 nm in diameter (Fig. 3*B*). Under these flow conditions, the fluidic microvortices induced by the herringbone-structured CNxCNT arrays, enhanced the interactions between the silica particles and the CNxCNT arrays during transport through a microfluidic channel (30). Notably, at flow rates that mimicked a manual push through a syringe, 4 × 10^3^ µL/min, the capture efficiencies could still reach ∼22%. To confirm that the size-based capture is facilitated by the presence of CNxCNT arrays, we assembled a microdevice without CNxCNT arrays as a control experiment. After passing the same mixture of the particles through the control arrays, very few particles (∼4%) were captured (*SI Appendix*, Fig. S4*C*), thus demonstrating the size range of the CNxCNT arrays.

We repeated the capture by reloading the microfluidic device into the same VIRRION via a manual push through a syringe to test whether the capture efficiency could be improved. The results indicated that the capture efficiency increased by ∼10% after the second capture (second capture in Fig. 3*D*). After the fourth capture, the efficiency increased another 10% on average, and the cumulative efficiencies were 42.4 ± 6.3%, 40.5 ± 5.4%, and 39.2 ± 6.1% for the 400-, 140-, and 25-nm silica particles, respectively (Fig. 3*C*). The control microdevice without CNxCNT arrays showed, as expected, low capture efficiencies (∼6%) for repeated capture under a flow rate of 250 µL/min (*SI Appendix*, Fig. S4*D*). These results demonstrate that the VIRRION can selectively capture different sizes of silica particles from a mixture, even at low flow-through pressure when using a syringe.

### Viable Virus Capture and Label-Free Detection.

**Fig. 4.**
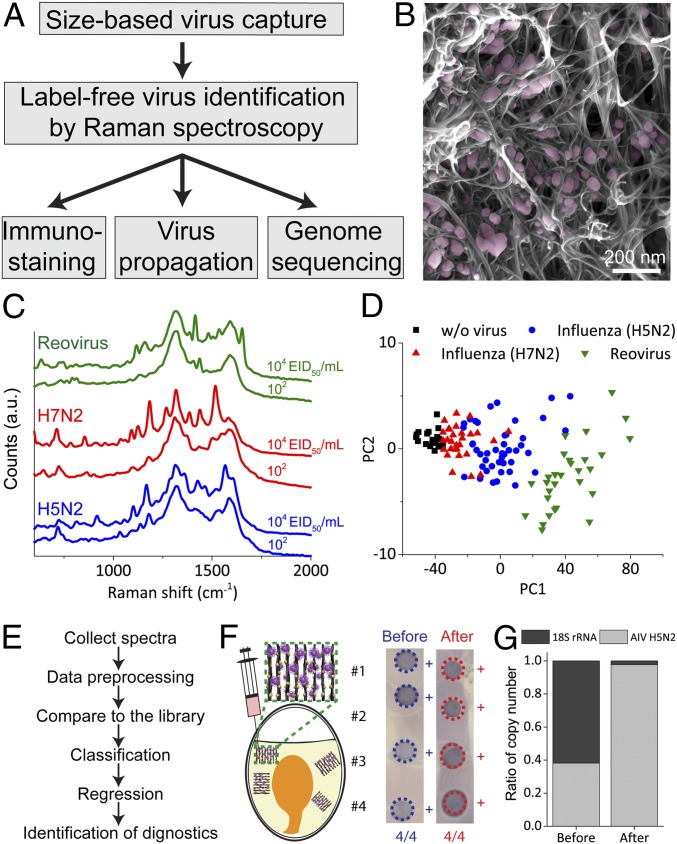
Characterization of avian influenza virus captured and detected by VIRRION. (*A*) A process flow of VIRRION for avian influenza virus surveillance and discovery. (*B*) SEM showing H5N2 virus particles captured by CNxCNT arrays. (*C*) Raman spectra of H5N2, H7N2, and reovirus collected from VIRRION. (*D*) Classification by PCA plot of Raman spectra collected from different avian viruses. (*E*) Process flow of virus identification by Raman spectroscopy with algorithm. (*F*) H5N2 virus propagated in ECE after viable capture and detection. (*G*) Ratio of copy number of H5N2 and 18S rRNA before and after VIRRION enrichment.

To test the efficacy of the VIRRION platform, we used a low pathogenic avian influenza A virus (LPAIV), which is an RNA virus (Fig. 4*A*). The average size of the H5N2 LPAIV is 101 ± 11.7 nm, measured by transmission electron microscopy (TEM) (*SI Appendix*, Fig. S5*A*). We assembled a VIRRION with an ITD of 100 nm to match the average size of the AIV. We manually pushed 5 mL of the supernatant from virus culture through a syringe containing 10^4^ EID_50_/mL (50% egg infective dose per microliter) H5N2 into a VIRRION. We then performed an immunofluorescence assay on the VIRRION to detect the captured AIV in situ by using an AIV H5 subtype-specific monoclonal antibody. Strong fluorescent signals were detected on the CNxCNT arrays (Fig. 1*B*), while no fluorescence was detected on the VIRRION when processing control samples that did not have H5N2 (*SI Appendix*, Fig. S5*B*). Under SEM and TEM, we clearly observed virus particles captured (Figs. 1*A* and 4*B*).

To test for in situ optical identification of the virus particles captured within the nanotube array via SERS, we developed an algorithm using a spectral database of 3 different viruses: strains from 2 AIV subtypes (H5N2 and H7N2) and reovirus. We first collected multiple Raman spectra after VIRRION capture of these viruses to build a simple database. Surface enhancement of the Raman scattering happens at a so-called “hot spot,” i.e., the 1-nm region between 2 adjacent gold nanoparticles. Thus, when viruses are trapped between the Au/CNxCNT arrays, the only Raman signal from the virus in the vicinity of the hot spot is enhanced. We recorded Raman signals and averaged over 100 spectra for each strain to generate a reliable average fingerprint for each virus. Each virus strain has a distinct fingerprint that was still distinguishable at concentrations as low as ∼10^2^ EID_50_/mL (Fig. 4*C*). It is noteworthy that this sensitivity is equivalent to that of RT-qPCR detection. In addition, no prominent peaks were detected in the negative control experiments (*SI Appendix*, Fig. S6). We applied a principal-component analysis (PCA) to classify all viral Raman spectra. Our results indicate that different strains cluster separately (Fig. 4*D* and *SI Appendix*, Note S1) (40). To develop a machine learning-based strategy to identify different virus strains, we used a 3-fold cross-validation to determine the best classification model out of 4 (logistic regression, support vector machine, decision tree, and random forest; Fig. 4*E*) (41–44). The logistic regression model minimizing multinomial loss achieved the highest validation accuracy (∼74%) in differentiating between H5N2, H7N2, Reovirus, and negative control (*SI Appendix*, Note S2). Since H7N2 and H5N2 have similar Raman spectra (Fig. 4*C*), there is a large overlapping region after classification, as shown in Fig. 4*D*.

### Virus Isolation and Enrichment.

After capturing and identifying H5N2 virus particles, we propagated the virus in cell culture directly on the VIRRIONs. Notably, during culture, the host cells attached and proliferated on the Au/CNxCNT arrays. The virus-like particles were observed on the surface of the cells, indicating effective replication and production of virus progeny (Fig. 1*B*). To confirm results of virus replication, we collected the supernatant and determined a virus titer of 10^6^ EID_50_/mL, which is similar to controls where the virus is propagated in the same cell line in a culture flask. We obtained a higher virus titer, ∼10^7^ EID_50_/mL, when we propagated captured viruses using embryonated chicken eggs (ECEs). To do that, we disassembled the VIRRION, collected the Au/CNxCNT with embedded viruses, and directly inoculated these into ECE for AIV propagation. For both cell culture and ECE propagation, we also confirmed viral replication by Dot-ELISA (26) (Fig. 4*F* and *SI Appendix*, Fig. S7*A*). These results clearly show that VIRRION maintains viability of the viruses during capture and makes direct virus isolation on-chip possible.

To demonstrate the efficiency of enrichment of the VIRRION array and the reduction of host nucleic acid during the enrichment, we targeted the 18S ribosomal RNA (rRNA), an essential housekeeping gene that is conserved in all eukaryotic cells. We used H5N2 spiked-in samples and extracted total RNA from the VIRRION after capture. We then characterized the enrichment by measuring the ratio of the copy number of host 18S rRNA to the virus hemagglutinin (HA) gene of H5N2 by RT-qPCR (*SI Appendix*, Fig. S7*B*) (27, 45). Before enrichment, the original ratio of 18S rRNA to H5N2 viral RNA was 1:0.61. After enrichment, the ratio was 1:42.47 (18S rRNA to H5N2), thus illustrating that H5N2 is significantly enriched by ∼69× (Fig. 4*G*).

We further analyzed the captured viruses by NGS to characterize the captured virus population without prior knowledge of the strains (14, 20–22, 46). We captured a mixture of samples containing more than one strain of viruses. When sequencing this type of sample, it is difficult to enrich without introducing bias (47, 48). We prepared samples by spiking equal volume, 0.2 mL, of H5N2 and H7N2 with an HA titer of 2^9^ (27), into viral transport media, and ran the pooled viruses on VIRRIONs consisting of one array of 100-nm ITD. After enrichment, the sequence data covers 8 complete genomic segments for each strain (*SI Appendix*, Fig. S8). We compared viral reads of the HA gene before and after enrichment. Interestingly, before enrichment, the H5N2:H7N2 ratio based on HA viral reads was 1:7.8, where 344 reads were from H5N2 and 2,693 reads from H7N2. After enrichment, the ratio was relatively well preserved at 1:6.5, but with an increase in viral reads (1,314 reads for H5N2 and 8,568 reads for H7N2). There appeared to be a more than 3-fold enrichment for both viruses. However, this difference in enrichment efficiencies maintained the stoichiometric proportion of viruses present in the original samples. Moreover, the complete genome of both strains was sequenced with an average coverage of 519× for H5N2 and 599× for H7N2. In addition, after enrichment, the host (chicken) reads decreased from 68.44% (reads) of the total reads (547,998 of 800,699 reads) to 34.2% (297,157 reads) of the total reads (866,855 reads). These results indicate that the VIRRION can minimize artifacts during enrichment. This feature is important for virus diversity profiling.

### Rapid Capture and Effective Identification of Human Respiratory Viruses.

**Fig. 5.**
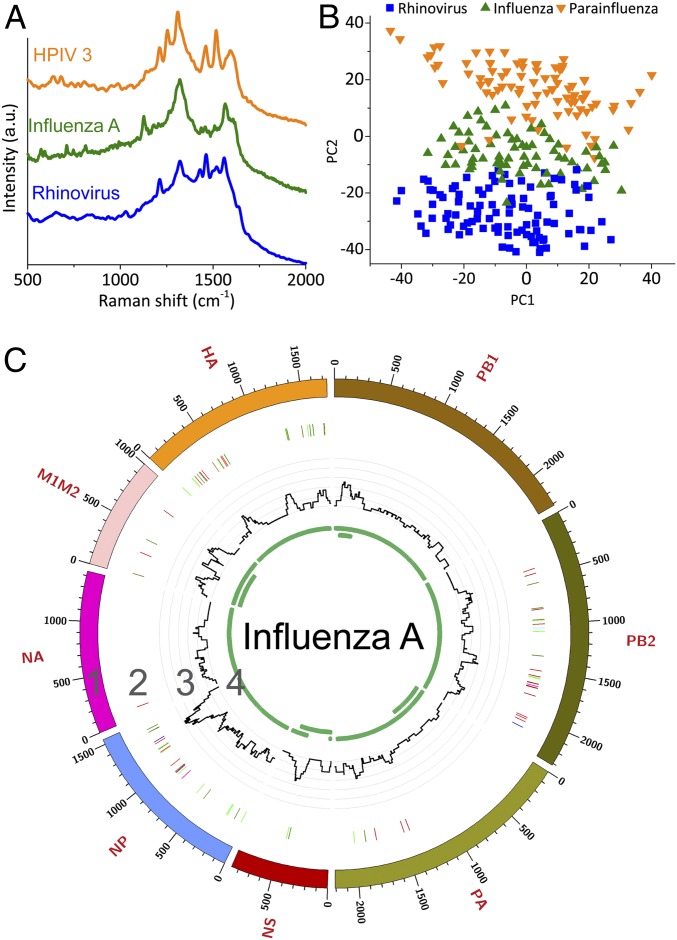
VIRRION testing of respiratory viruses. (*A*) Raman spectra. (*B*) PCA plot of Raman fingerprint of the different viruses. Each dot represents a collected spectrum. (*C*) Circos plots of coverage and variants of captured influenza viruses. Genome segment sequencing and analysis of influenza A mapped to strain A/New York/03/2016 (H3N2). Track 1: scale of the base pair position; track 2: variant analysis by mapping to strain H3N2 (KX413814–KX413821), color code: deletion (black), transition (A–G, fluorescent green; G–A, dark green; C–T, dark red; T–C, light red), transversion (A–C, brown; C–A, purple; A–T, dark blue; T–A, fluorescent blue; G–T, dark orange; T–G, violet; C–G, yellow; G–C, light violet); track 3: coverage; track 4: regions of ORF.

Acute respiratory infections are the third most common cause of death worldwide and responsible for 4 million deaths each year, which is 7% of all deaths annually (18). Most clinical or field samples have very low virus titers, and sequencing of total RNA usually results in a majority (>80% of the sequence reads) of host sequences, such as rRNA (20, 49). When developing methods for enrichment, the challenge is to minimize bias and to process clinically relevant sample volumes (12, 14, 20, 21). We validated the VIRRION in human respiratory infection diagnostics by rapidly capturing and identifying different viruses in nasopharyngeal swabs from patients who had been diagnosed with rhinovirus, influenza A virus, or human parainfluenza virus type 3 (HPIV 3). The diagnosis was confirmed by TEM and PCR (*SI Appendix*, Figs. S9 and S10) (50–58). We assembled a VIRRION with ITDs of 200, 100, and 30 nm, which cover the size range of most viruses that commonly cause respiratory infections. Without any sample preparation, we loaded 3 mL of the transport media in which the swabs were stored into separate VIRRIONs through a syringe and gently pushed manually. After capture, virus-like particles were observed by SEM on CNxCNT arrays (*SI Appendix*, Fig. S11). We extracted nucleic acid on-chip from the VIRRIONs and confirmed by RT-qPCR what viruses were captured (*SI Appendix*, Fig. S10). Their Raman spectra fingerprints were determined and recorded (Fig. 5*A*). The PCA (Fig. 5*B*) demonstrated that the Raman spectra could clearly differentiate between the virus strains when converting the data into a low-dimensional (2D) scale. Next, we applied the previously developed machine learning strategy to classify the results of the Raman spectroscopy. The accuracy for identification of the specific viruses was ∼93% using the logistic regression algorithm (*SI Appendix*, Notes S1 and S2). Compared to conventional detections, such as ELISA or PCR, our results indicate that VIRRION can be successfully used to detect specific viruses within several minutes after collecting clinical samples.

After label-free capture and detection, we processed the isolated nucleic acid for NGS and determined that after VIRRION capture, the influenza A and HPIV 3 viruses were significantly enriched (*SI Appendix*, Figs. S13 and S14 and Table S1). The percentage of virus-specific reads increased from 0.08 to 0.44% for influenza A and from 4.1 to 31.8% for HPIV 3. Genome coverage also increased following enrichment. The percentage of the influenza A genome increased from 33% before enrichment to 48% and 57% following the first and second enrichment steps, respectively. The percentage of the HPIV 3 genome increased from 22% before enrichment to 81% and 77% after the first and second enrichment steps. The average genome coverage increased from 8.8× to 15.9× for influenza, and 363× to 2,040× for HPIV 3. We were also able to assemble large portions of each viral genome. No influenza A contigs greater than 500 bp were identified following assembly in the unenriched sample. However, 4 contigs matching the influenza A genome were found in both enrichment steps with an average of 590 bp. They are 681-bp matches to PB1 (MH540988.1), 622-bp matches to PB1 (MH845979.1), 570-bp matches to NA (LC409054.1), and 553-bp matches to PB2 (MH540997.1). The entire genome was assembled in each of the HPIV 3 samples. The results of coverage and variant analyses were summarized in Fig. 5*C*. For HPIV 3, we mapped the reads against strain H7N2 (MF973163.1), as shown in Fig. 1*B* and *SI Appendix*, Fig. S12. The NGS results supported that VIRRION can enrich different viruses directly from patient swab samples. Unfortunately, no rhinovirus-related reads were detected by NGS after enrichment. We suspect that this is because the virus titer is extremely low even after VIRRION enrichment, as determined from PCR results (*SI Appendix*, Fig. S10).

In summary, our newly developed VIRRION provides a multifunctional and portable microplatform for rapid virus capture and sensitive in situ identification by SERS. Currently, we are expanding the Raman database by collecting more spectra from different types of viruses. An expanded database would allow better characterization of unknown viruses. The captured viruses are viable and enriched, thus providing effective sample preparation for existing standard methods for virus analysis, including cell culture for virus isolation, immunostaining, and NGS. We also successfully captured and detected different human respiratory viruses from clinical samples using this platform. Two of its strengths are that it can perform enrichment in just a few minutes and achieve a sensitivity comparable to that of RT-qPCR with 70∼90% accuracy. This platform provides a way to overcome the technical barrier in virus surveillance and discovery, and its many salient features would also help in virus prediction and outbreak preparedness.

## Materials and Methods

Precursor solutions were prepared by diluting Fe (NO_3_)_3_•9H_2_O (Sigma-Aldrich; #254223-10G) using a mixture (1:1) of DI water and toluene (Sigma-Aldrich; #244511-100 ML). To perform stamping, a PDMS-coated mold was placed on a silicon substrate covered with a thin film of liquid iron precursors for 30 s. Then, the PDMS stamp was transferred and applied on a fused silica substrate for 10 min in order to ensure iron precursors were transferred and dried. More detailed information regarding the materials and methods are available in *SI Appendix*. All of the raw NGS data have been deposited in the Sequence Read Archive (SRA) at the National Center for Biotechnology Information under submission number SUB5200307.

## Supplementary Material

Supplementary File
