# Supplementary material for: A rapid and label-free platform for virus capture and identification from clinical samples
Source: Proc Natl Acad Sci U S A. 2019 Dec 27;117(2):895–901. doi: 10.1073/pnas.1910113117 (PMC6969489; doi:10.1073/pnas.1910113117)
Supplement: Supplementary File [file pnas.1910113117.sapp.pdf]

Supporting appendix

A rapid and label-free platform for virus capture and identification from clinical samples

Yin-Ting Yeh[a], Kristen Gulino[b], YuHe Zhang[a], Aswathy Sabestien[c], Tsui-Wen Chou[b], Bin Zhou[b], Zhong Lin[a], Istvan Albert[c], Huaguang Lu[d], Venkataraman Swaminathan[a], Elodie Ghedin[b], Mauricio Terrones[a]

[a] *Department of Physics, The Pennsylvania State University, PA 16802, USA*

[b] *Department of Biology, New York University, NY 10003, USA*

[c] *Department of Biochemistry and Molecular Biology, The Pennsylvania State University, PA 16802, USA*

[d] *Department of Veterinary and Biomedical Sciences, The Pennsylvania State University, PA 16802, USA*

Corresponding Authors:

Yin-Ting Yeh, Ph.D.

205 Osmond

The Pennsylvania State University, University Park, PA 16802

Tel: +1-814- 303-9885

Email: yxy155@psu.edu

Mauricio Terrones, Ph.D.

205A Osmond

The Pennsylvania State University, University Park, PA 16802

Tel: +1-814-865-0343

Email: mut11@psu.edu

www.pnas.org/cgi/doi/10.1073/pnas.1910113117

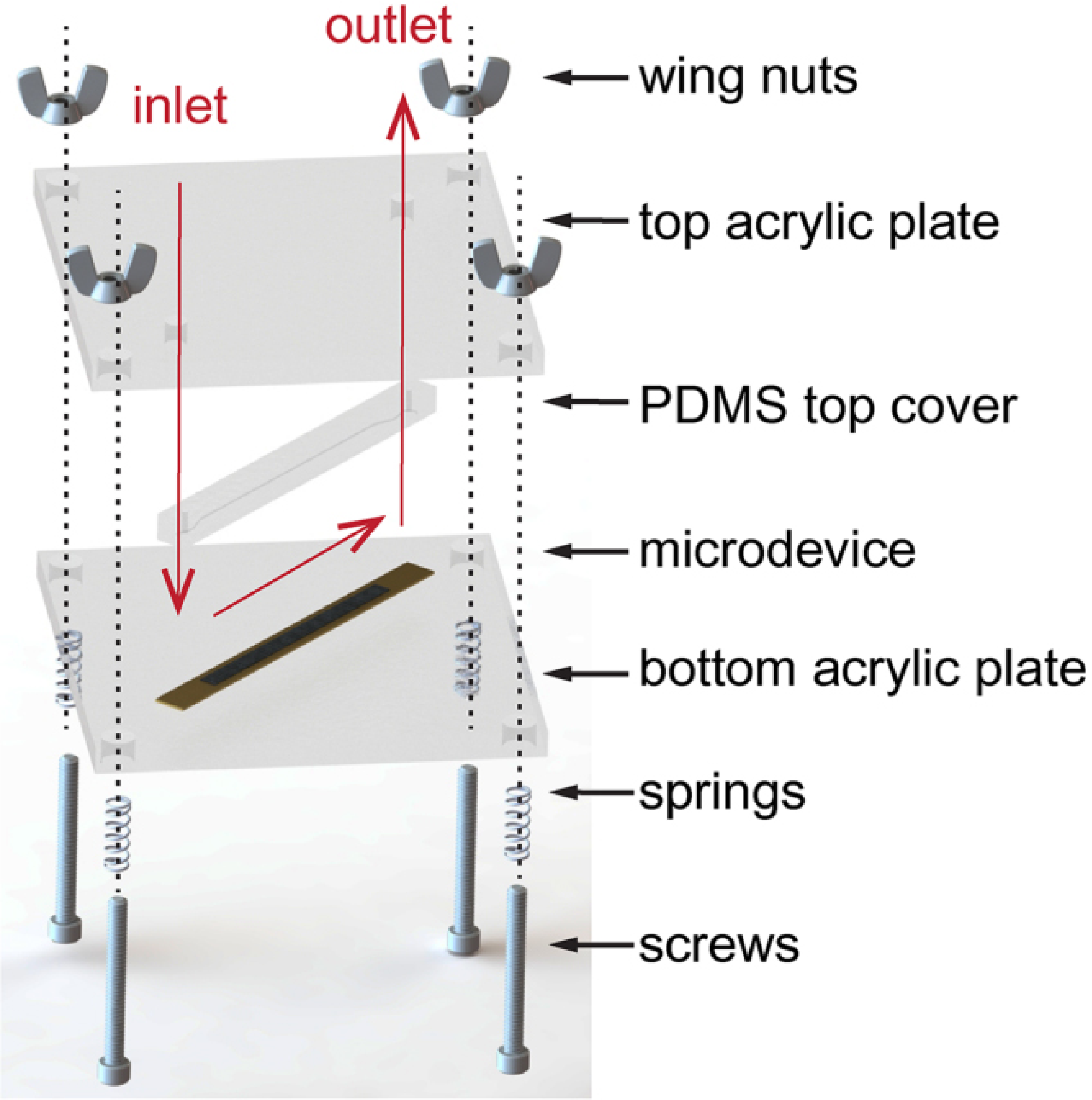

Supplementary Figure 1. Illustration of disassembled view of VIRRION components.

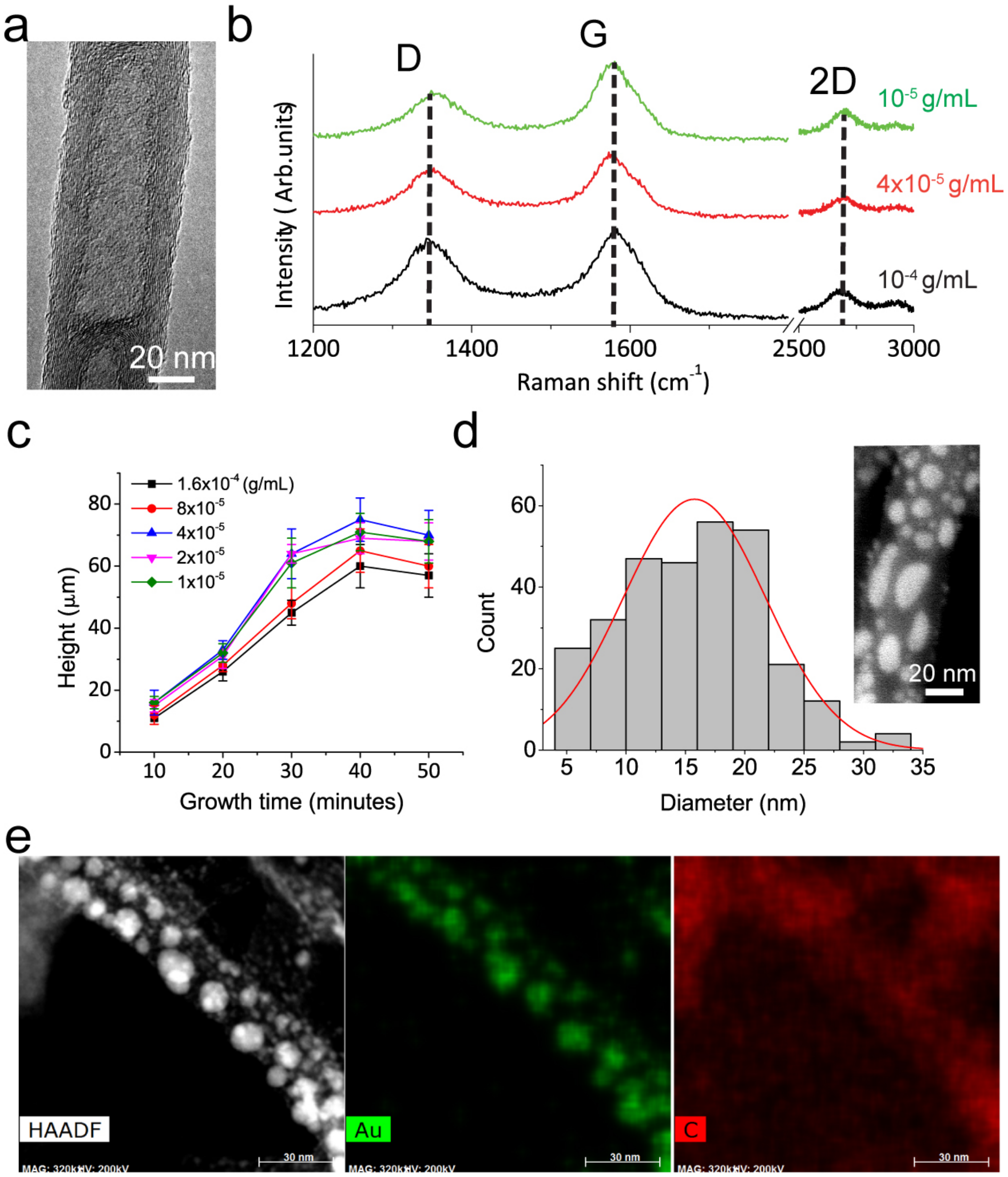

Supplementary Figure 2. Characterization of CNxCNTs decorated with gold nanoparticles. a) TEM image of CNxCNT. b) Raman spectra with signature peaks of nitrogen-doped carbon nanotube. c) Height of the aligned CNxCNTs synthesis with different concentrations of precursors and synthesis time. d) Histogram of gold nanoparticles decorated on CNxCNT. The average diameter of gold particles is 15.6 ± 6nm. e) TEM and Energy-dispersive X-ray spectroscopy (EDX) of the CNxCNT decorated with gold nanoparticles.

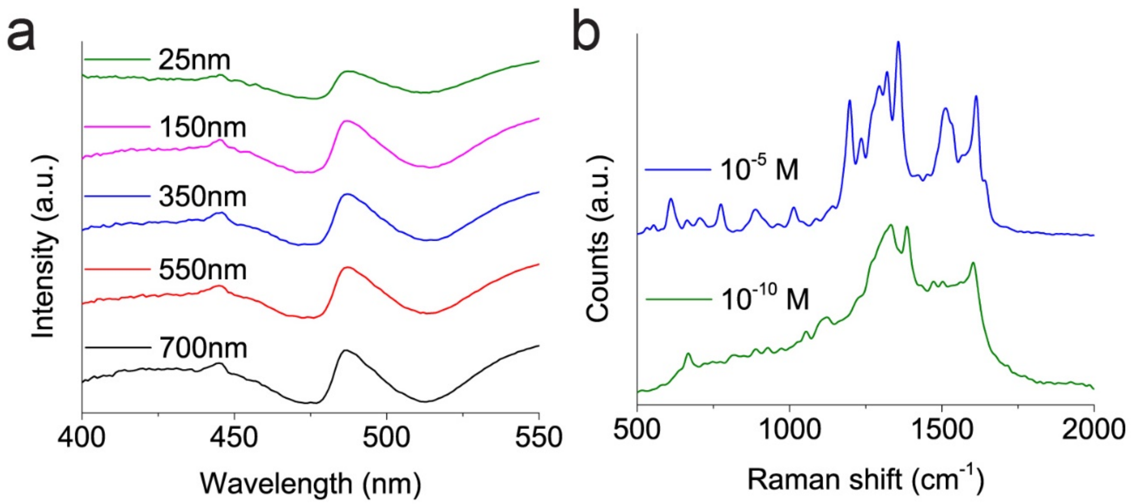

Supplementary Figure 3. Absorption and Raman spectroscopy of Au/CNxCNTs. a) Absorption spectra of Au/CNxCNT arrays with different ITDs measured by UV-Vis. b) Raman spectra of Rhodamine 6G measured by Au/CNxCNT arrays.

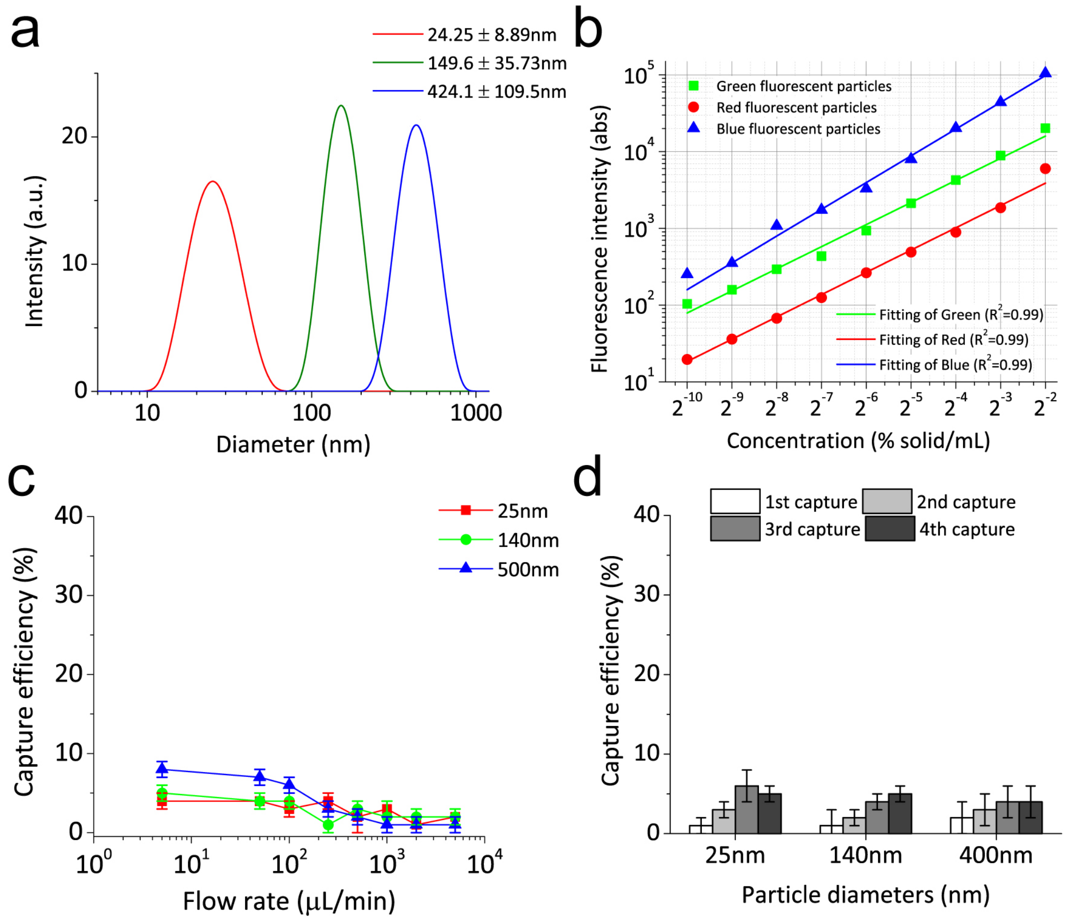

Supplementary Figure 4. Characterization and standard curve of fluorescently labelled particles and capture efficiency of VIRRION without CNxCNTs (control). a) Histogram of diameters of fluorescent particles. b) Standard curve of fluorescence intensity as a function of particle concentrations; abs=absorbance. c) Capture efficiency of VIRRION without CNxCNTs (control) under different flow rates. d) Capture efficiency of VIRRION without CNxCNTs (control) under 250 μL/min of repeated capture.

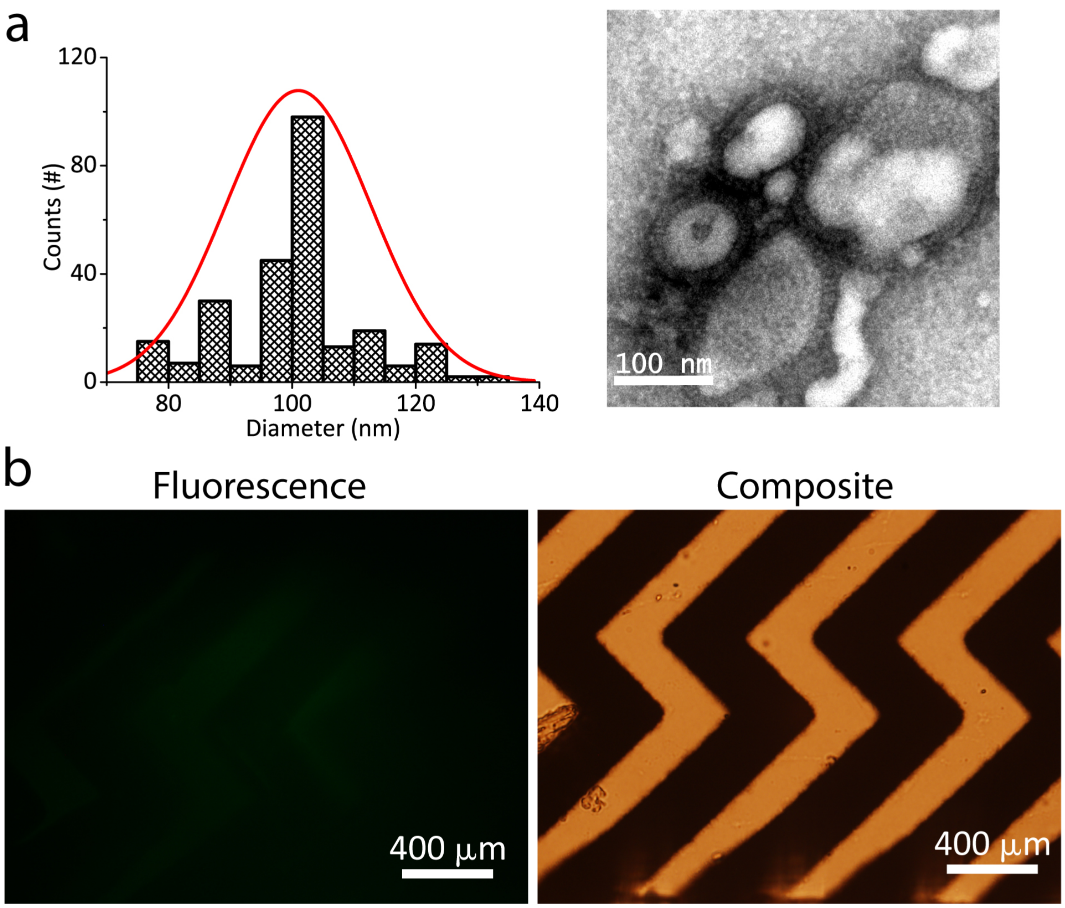

Supplementary Figure 5. Avian influenza virus H5N2 captured by VIRRION. a) Histogram of H5N2 diameter and TEM image of H5N2. b) Immunostaining of media without H5N2 (control).

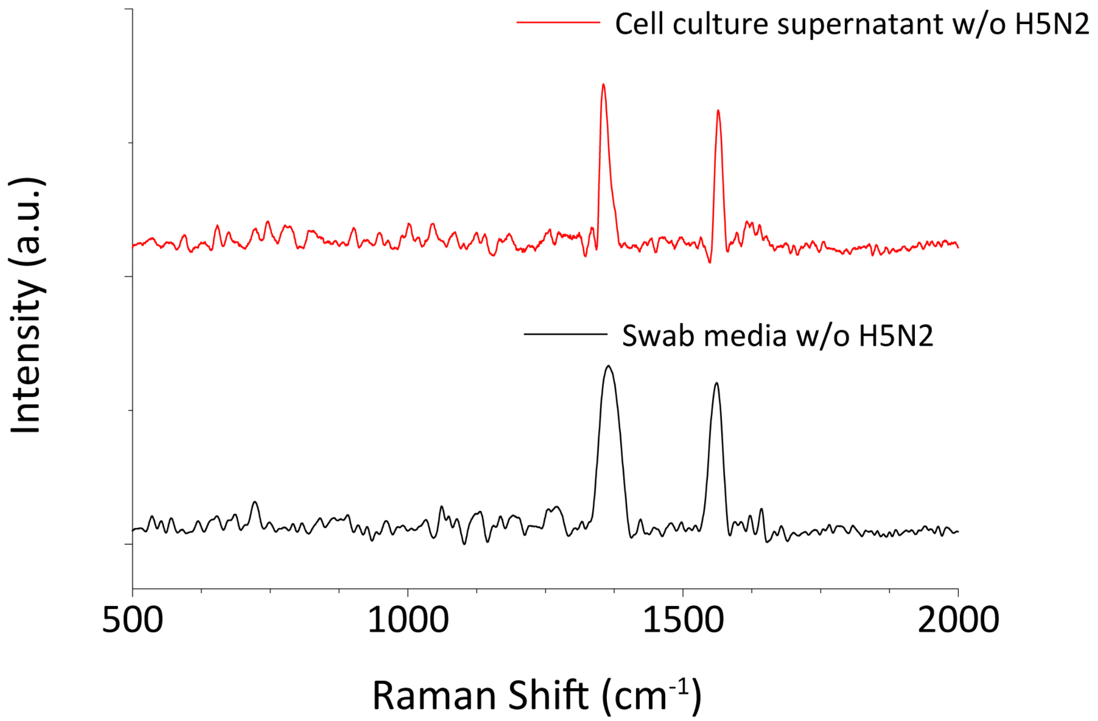

Supplementary Figure 6. Raman spectra of cell culture supernatant and swab media without H5N2 virus (negative control).

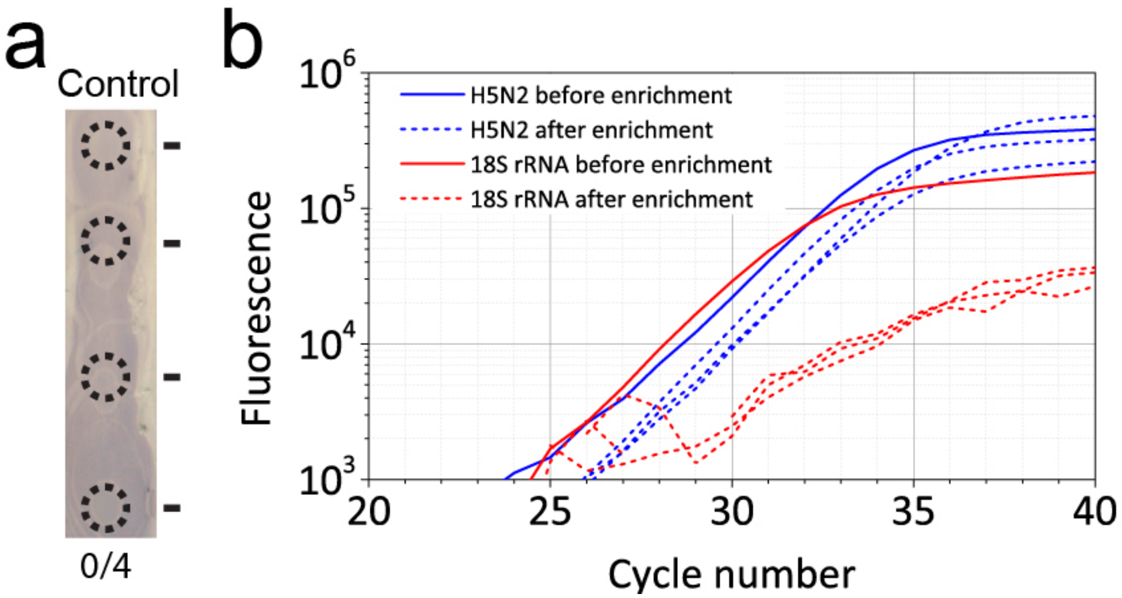

Supplementary Figure 7. Control experiment of viable VIRRION capture and enrichment. a) Results of Dot-ELISA for CNxCNT structure without viruses through egg propagation. b) RT-qPCR results of H5N2 and 18S rRNA before and after VIRRION enrichment.

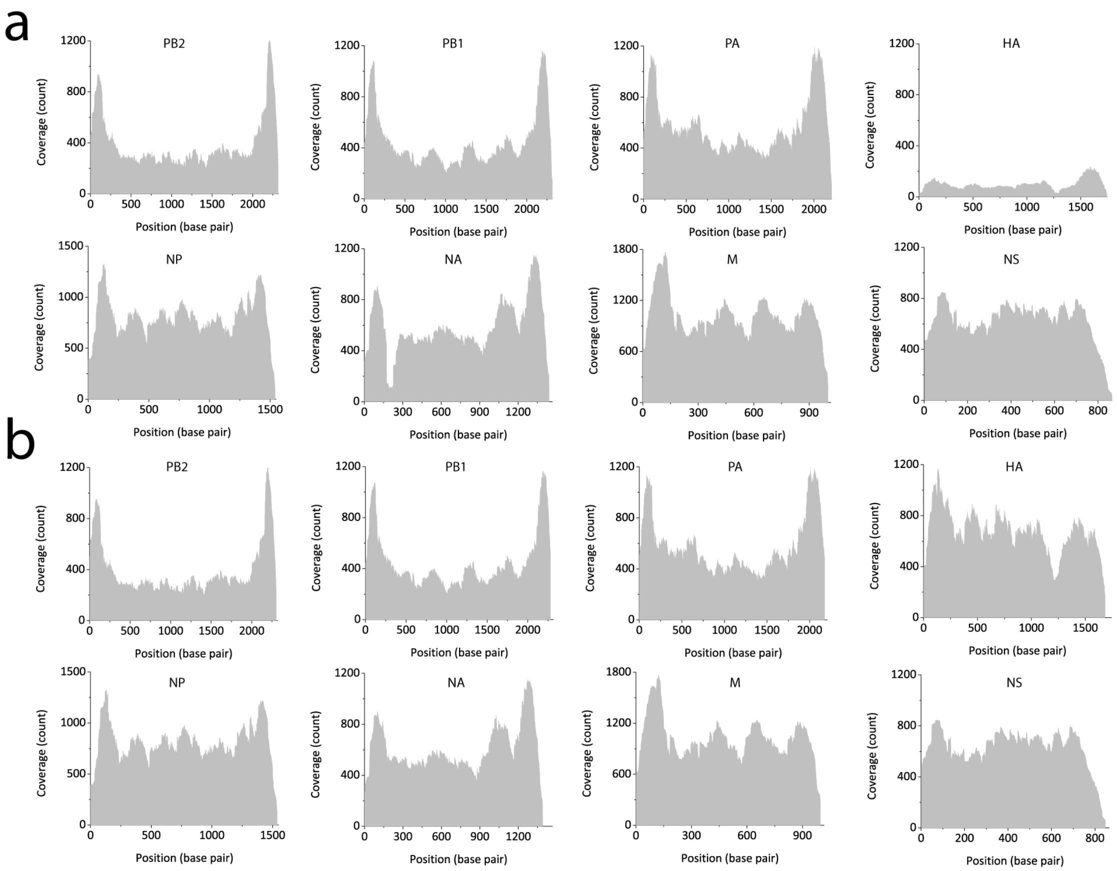

Supplementary Figure 8. Coverage plot of mapped avian influenza virus reads on genomic segments after capture and enrichment by VIRRION. a) H5N2. b) H7N2. HA=hemagglutinin; NA=neuraminidase; PB1, PB2, PA = polymerases; NP=nucleoprotein; M= matrix; NS= non-structural protein.

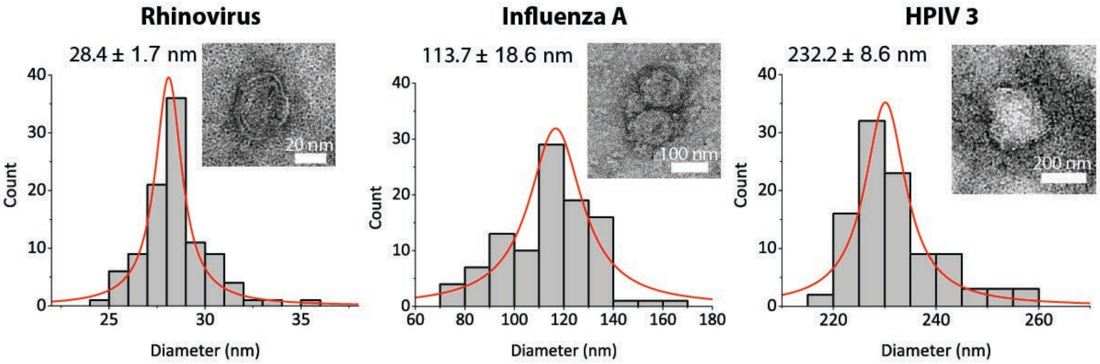

Supplementary Figure 9. TEM images and histograms of sizes of Human viruses. a) Rhinovirus. b) influenza type A. c) parainfluenza type 3.

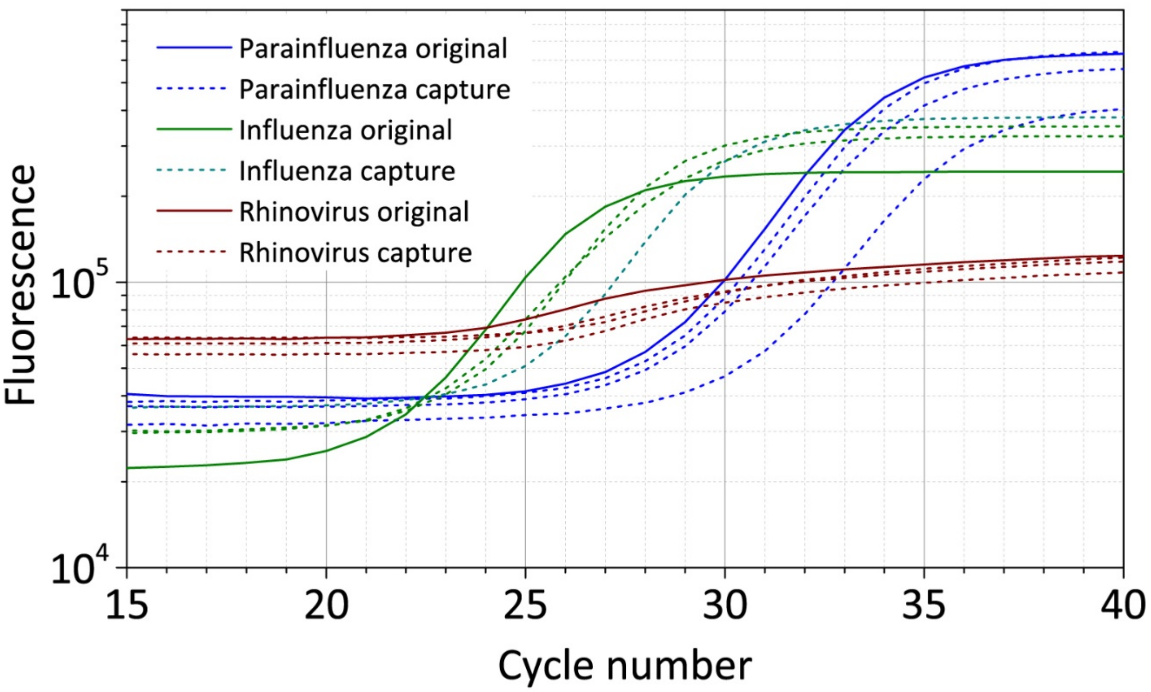

Supplementary Figure 10. RT-qPCR results of respiratory viruses before and after VIRRION capture.

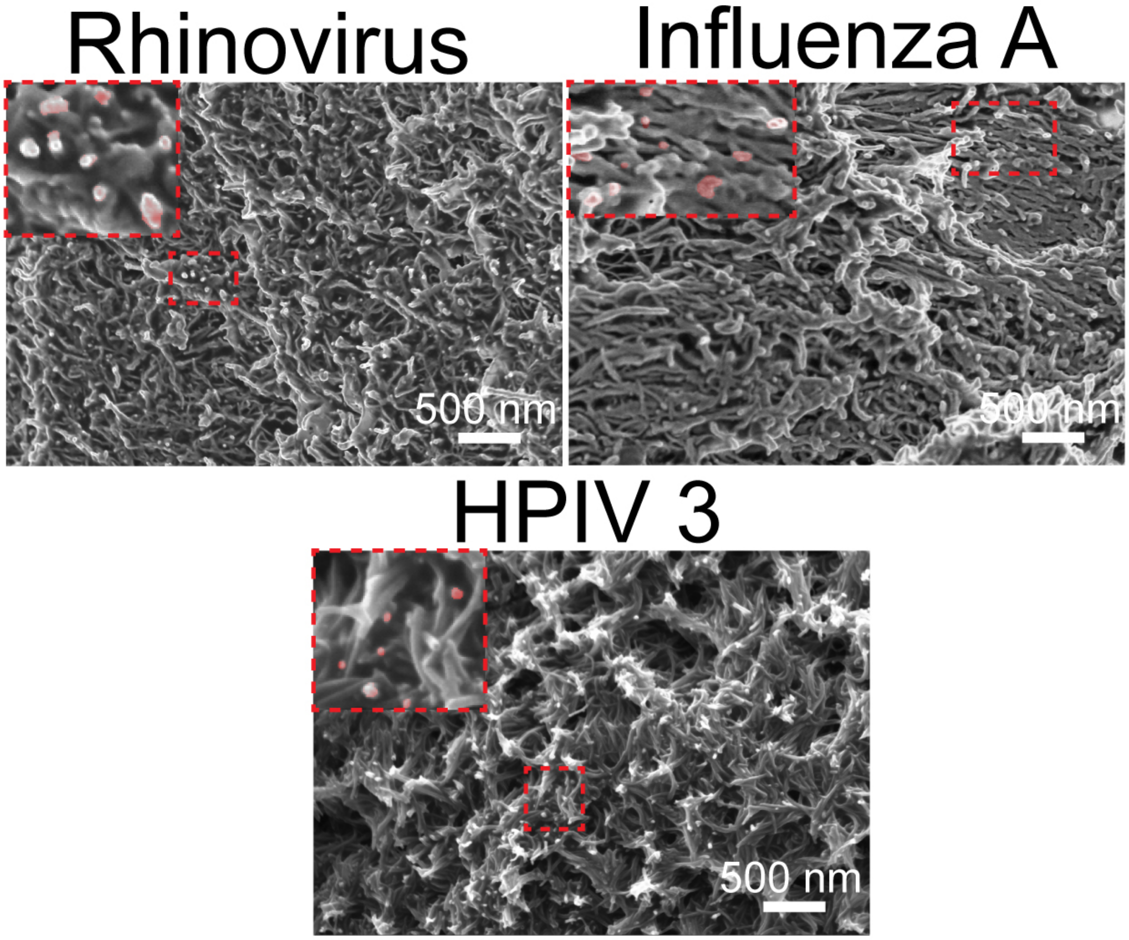

Supplementary Figure 11. SEM images of different respiratory virus-like particles captured by VIRRION.

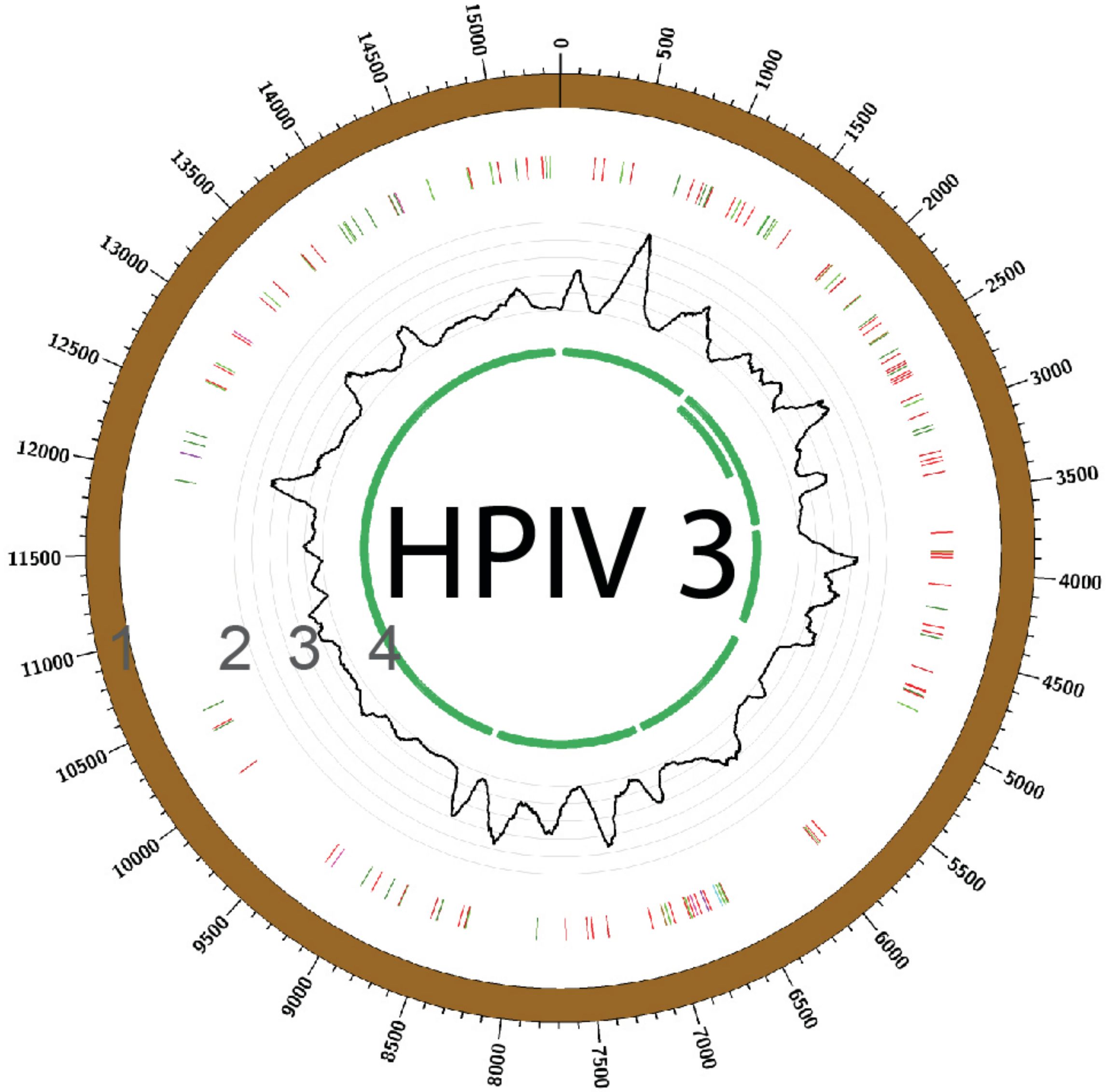

Supplementary Figure 12. Genomic sequencing and analysis of human parainfluenza type 3 (HPIV). track 1: scale of the nucleotide position; track 2: variant analysis by mapping to strain #MF973163, color code: deletion (black), transition (A-G, fluorescent green; G-A, dark green; C-T, dark red; T-C, light red), transversion (A-C, brown; C-A, purple; A-T, dark blue; T-A, fluorescent blue; G-T, dark orange; T-G, violet; C-G, yellow; G-C, light violet).; track 3: coverage; track 4: regions of open reading frame.

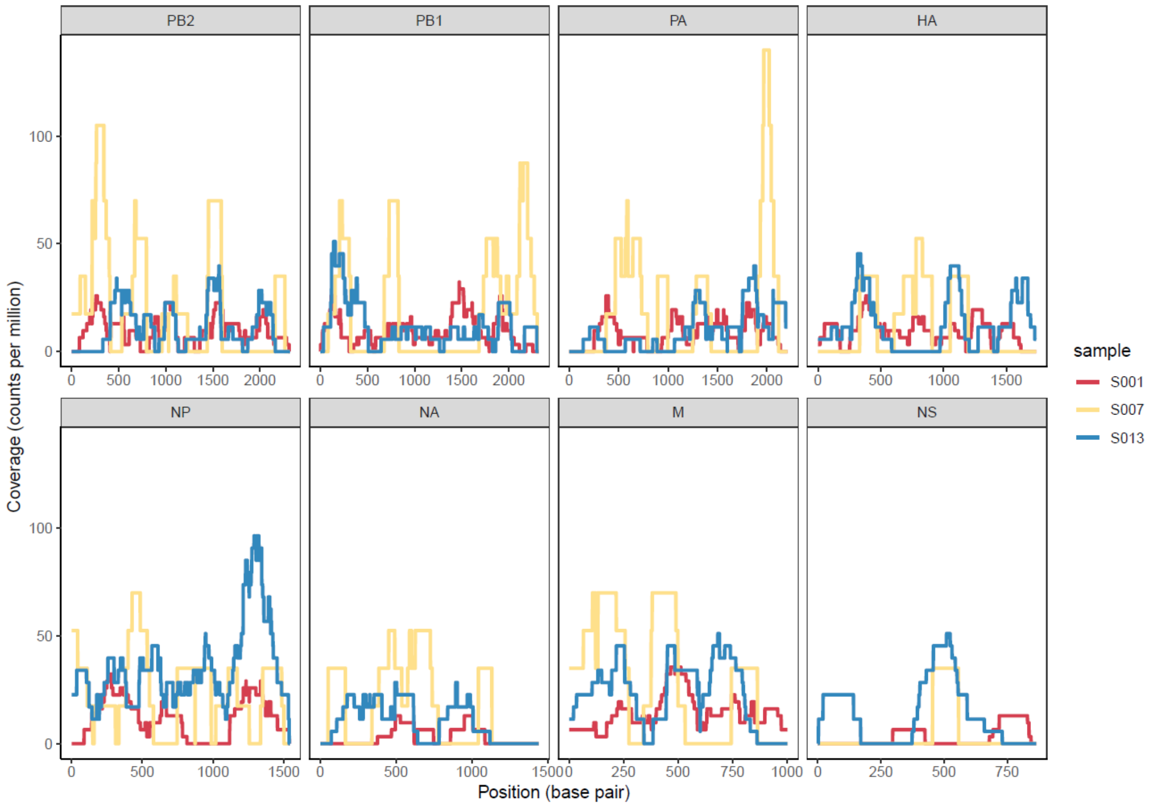

Supplementary Figure 13. Coverage of influenza virus segments after virus capture and mapping of NGS reads to a reference strain (A/New York/03/2016(H3N2)). S001 represents the sample before enrichment, S007 represents the sample after one round of enrichment, and sample S013 represents the sample after two rounds of enrichment.

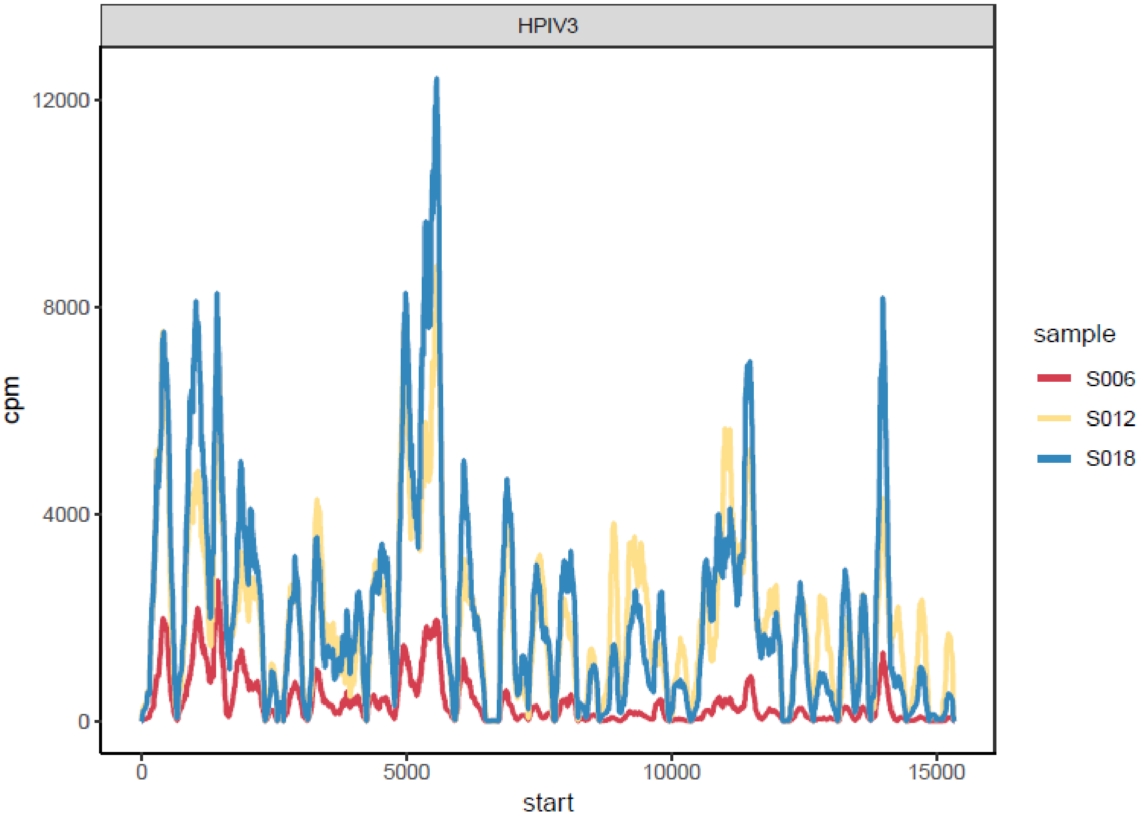

Supplementary Figure 14. Normalized coverage of human parainfluenza type 3 genome after virus capture and mapping of NGS reads to reference strain MF973163. S006 is the unenriched sample, S012 is the sample following one enrichment step, and S018 is the sample following two enrichment steps.

Supplementary Note 1

Principal component analysis (PCA) is a statistical technique that reduces the dimension of a dataset to visualize data set with higher dimensions (41). Specifically, PCA finds a new coordinate system so that the projected data on the first coordinate (called the first principal component) shows the most significant variance of the data, and projection on the second coordinate shows the second most significant variance, and so on. Each spectrum of our Raman data contains 1932 data points and is treated as a 1932-dimensional vector. In order to visualize the spectra of different viruses to see if the Raman spectra can capture the differences and variances, we use PCA to reduce the dimension of the spectra data. We first standardize the spectra data so that the mean value equal to zero and standard deviation equal to one in each dimension. We then apply PCA to project each spectrum on the first and second principal components. The first two principal components explain more than 90% of the variance in the whole dataset. In this way, each spectrum is represented by a point on a 2D coordinate system. Given all the 2D data points of one type of virus, we remove the outliers with a fraction of 0.2 using a standard method called Elliptic Envelope. Figure 4.d plots all the inliers of the 2D points which clearly shows the differences and variations between samples from different viruses.

Supplementary Note 2

Through PCA, we visualize the dimension-reduced Raman spectra data points on a 2D plot, which indicates that data points from different viruses are clearly separable. Next, we apply machine learning (ML) methods to classify the Raman spectra generated by different virus samples. It is a typical classification problem, for which the input is a Raman spectrum and the output is a virus category. The most common ML algorithm for such classification problems includes Logistics Regression (43), Support Vector Machine (42), Decision Tree (44) and ensemble methods of Decision Tree, such as Random Forest (45). We test these four methods on our dataset with 3-fold cross-validation: first split the whole dataset into 3 groups evenly; then, for each group, hold it as a validation set and train the model on the remaining data; finally evaluate the accuracy of the trained model on the validation set. The average accuracy on the hold-out validation set is used to select the best model. We find the Logistic Regression produces the highest mean validation accuracy (~90%), where Support Vector Machine (~78%), Decision Tree (~70%) and ensemble methods of Decision Tree (~83%) on respiratory virus samples. We also tested if preprocessing the data by standardization, the same preprocessing used in PCA, improves the accuracy. We found that the results almost remain the same for Logistic Regression, so no preprocessing is needed.

Materials and Methods

Patterning and growth of the CNxCNT

A mold with a pattern for stamping was designed by Solidwork (v2018) and manufactured by a 3D printer (Flashforge Creater Pro) using polylactide (PLA) as a building material. We coated molds with uncured polydimethylsiloxane (PDMS) and cured overnight for PDMS crosslinking. Then, 100 μL of the precursor solution was spun on a silicon substrate (2cm by 2cm) with 300 nm thick oxide layer under 500 rpm for 5 seconds and followed by 1000 rpm for 30 seconds. Before stamping, both PDMS-coated stamps and glass substrates were treated by a mild air plasma. The growth and the characterization of the CNxCNTs were described in our previous report (29). After CVD growth, gold was deposited on the CNxCNT array by using sputter (Kurt J. Lesker Lab-18) and followed by annealing at 400°C for 10 minutes. Image analysis was performed using a field emission SEM (LEO 1530 FESEM) to measure dimensions through cross-sectional images.

The efficiency of size-based capture

Fluorescently labeled particles were purchased from Thermo Fisher Scientific (Fluoro-Max Dyed Aqueous Fluorescent Particles) and diluted with DI water. Fluorescence intensity was measured by a microplate reader (Tecan Infinite F200). Capture efficiency was calculated by comparing the fluorescence intensity of the original sample to the flow-through samples using standard curves (Sup. Fig.4).

Raman spectroscopy

Rhodamine 6G (Sigma Aldrich, Cat# 252433) was diluted with ethanol and then applied to the Au/CNxCNT substrate. A substrate was immersed in R6G solution for 5 minutes and then rinsed by pure ethanol solution. For virus samples, VIRRION was disassembled and measured by Raman spectroscopy. Raman spectra were recorded by Raman microscopy (Renishaw, InVia Raman microscopy) using 488 nm or 785 nm lasers for 30 seconds under 50X magnification with 10 μW of the laser power.

Virus sample preparation

We used a low pathogenic AIV (LPAIV) subtype H5N2 and H7N2, and spiked it into viral transport media (BD, #220531) to mimic a swab sample. The avian virus was propagated in specific pathogen-free (SPF) embryonated chicken eggs (ECE) via allantoic cavity route inoculation at 9-11 days of age. The inoculated eggs were placed in a 37°C egg incubator for 72 hours. The eggs were then removed from the incubator and chilled at 4 °C for 4 hr. Each egg was cracked open at the top air sac and the shell peeled without breaking the air sac membrane, and allantoic fluid containing the virus was harvested using a 3 mL sterile syringe with a 25G×5/8" needle. The harvested allantoic fluid was clarified by centrifugation at 8000 rpm for 5 minutes. The virus titers were measured in embryo infectious doses 50% (EID_{50}) by the Reed-Muench method. Briefly, the EID_{50} test was conducted in ECE. The propagated fresh stock H5N2 AIV was prepared in 10-fold serial dilutions from 10^1 through 10^9. Each dilution was inoculated into 5 eggs, 0.1 mL per egg. The inoculated eggs were incubated at 37 °C for 72 hours. After 72 hours of incubation, allantoic fluid was harvested from each egg. The infection status of each egg was determined by Dot-ELISA (27).

On-Chip cell culture and virus AIV propagation

After capturing AIV, VIRRIONs were disassembled and placed into petri dishes (Falcon™ Standard Tissue Culture Dishes, Corning 353002) for cell culture. We then applied a chicken hepatocellular carcinoma cell line (LMH; ATCC CRL-2117) directly onto the CNxCNT arrays. A standard cell culture procedure, as instructed by the manufacture, was followed. For virus propagation after capture, viruses were embedded within the AU/CNxCNT structure and we disassembled the VIRRION. We used a pipette tip to harvest the Au/CNxCNT structure and transferred the tips to a centrifuge tube containing virus transport media (Becton Dickinson Cat#220244). We followed the virus propagation procedure previously described.

Sample preparation for Electron microscopy

For SEM sample preparation, samples were fixed with 4% paraformaldehyde and were then dehydrated with ethanol under a serial dilution from 50% to 100% (pure ethanol). For TEM, samples were dropped onto Quantifoil Copper grids treated with a mild air plasma (PELCO easiGlow™ Glow Discharge Cleaning System) and then negative staining was applied.

On-chip immunostaining

After virus capture, phosphate buffered saline (PBS) was introduced to flush out the inside of the VIRRION. We disassembled the VIRRION and applied monoclonal antibody against the H5 HA protein (100 µL of 1:1000 diluted work solution, Penn State ADL). After incubation at 37°C for 40 minutes, we washed the substrate with 1 mL of PBS and applied goat anti-mouse immunoglobulin conjugated with FITC (100 µL of 1:500 work dilution, KPL). After another incubation at 37 °C for 40 minutes, we flushed the substrate with PBS to remove any non-specific binding. Fluorescence microscopic images were obtained by imaging the device directly with a fluorescence microscope (Olympus IX71).

Respiratory samples

Nasopharyngeal swabs collected and stored as part of a separate IRB-approved study at the Hershey Medical Center (PA, USA) of patients with respiratory infections were provided for VIRRION analysis. Three samples were available from patients diagnosed with Influenza A virus, HPIV-3, or Rhinovirus. The nasopharyngeal swabs were collected using the BD swab collection kit (BD, # 220527) and stored in a -80ºC refrigerator prior to use. The storage media is what was used on the VIRRION, without dilution or pre-processing.

Viral nuclei acid extraction and quantitative PCR detection

After virus capture, we applied 300 µL lysis solution (Qiagen, QIAamp MinElute Virus Spin Kit) into the microdevice and collected supernatants. The viral RNA was extracted with QIAamp MinElute Virus Spin Kit (Qiagen) following the manufacturer's protocol. RT-qPCR detection was performed with a real-time PCR system (Applied Biosystem Inc. 7300) using thr QIAGEN OneStep RT-PCR Kit (Cat# 210212). The primers used for for RT-qPCR were for influenza A (52): AAAGCGAATTTCAGTGTGAT (sense), GAAGGCAATGGTGAGATTT (antisense); influenza B (52): GTCCATCAAGCTCCAGTTTT (sense), TCTTCTTACAGCTTGCTTGC (antisense), RSV(52): TTTCCACAATATYTAAGTGTCAA (sense), TCATCWCCATACTTTTCTGTTA (antisense); HPIV 2 (52): CCATTTACCTAAGTGATGGAA (sense), CGTGGCATAATCTTCTTTTT (antisense); HPIV 3(52): GGAGCATTGTGTCATCTGTC (sense), TAGTGTGTAATGCAGCTCGT (antisense); and Rhinovirus (53): GGGTGYGAAGAGYCTANTGTGCT (sense), GGACACCCAAAGTAGTYGGTYC (antisense)

NGS sample preparation and analysis

Total RNA of the samples was extracted and reverse transcribed into cDNA by TruSeq Stranded Total RNA Sample Prep Kit (Illumina, San Diego, CA). We followed the manufacturer's instruction but skipped poly-A enrichment. The quality of the cDNA library was characterized by the Agilent Bioanalyzer system (Agilent Technologies, Santa Clara, CA, USA). Library concentration was assessed by qPCR using the KAPA Library Quantification Kit Illumina Platforms (Kapa Biosystems, Wilmington, MA, USA). Finally, the prepared cDNA libraries were loaded into different lanes of the MiSeq sequencer using 150 nt single read sequencing (Illumina, San Diego, CA, USA).

Sequencing analysis

We removed the adaptors and performed quality trimming using Trimmomatic (54). We used DeconSeq (55) to remove reads that matched to the human reference genome. The remaining reads were mapped to reference strains using Bowtie2 (56). LASTZ and SAMtools (57, 58) were used to identify and obtain the final virus consensus sequences. Intrahost variants (iSNVs) were identified using a haplotype-based variant detector (Freebayes) with a setting of ploidy of 1 and error rate of 1% for Illumine MiSeq. If the frequency of the variant population was higher than

1%, it was considered an iSNV site. The genetic variants were annotated by SnpEff v4.1. The NGS data was displayed by generating plots with Circos (v 0.67). Read coverage was analyzed by mapping reads to reference strains using Bowtie2. To normalize between library sizes, counts per million were calculated. Genome assembly was performed *de novo* using metaSpades (59) and contigs > 500 bp were retained for analysis. These contigs were assigned taxonomy by using blastn and the NCBI nt database. They were further confirmed using the viral RefSeq database provided by NCBI.

Data Availability Statement: All data discussed in the paper will be made available to readers. All the raw NGS data were upload to Sequence Read Archive (SRA) at NCBI under submission number SUB5200307.
